# Invasive fungal infection in patients with hematologic malignancies: epidemiology and prognostic factors

**DOI:** 10.11604/pamj.2024.48.130.40509

**Published:** 2024-07-24

**Authors:** Maroua Jebari, Latifa Mtibaa, Hela Ghedira, Nawel Baccouchi, Sami Zriba, Fehmi Msadek, Boutheina Jemli

**Affiliations:** 1 Laboratory of Parasitology-Mycology, Military Hospital of Tunis, Tunis, Tunisia,; 2 Faculty of Medicine of Tunis, University Tunis El Manar, Tunis, Tunisia,; 3 Department of Hematology, Military Hospital of Tunis, Tunis, Tunisia,; 4 Faculty of Pharmacy of Monastir, University of Monastir, Monastir, Tunisia

**Keywords:** Invasive fungal infection, hematologic malignancy, epidemiology, prognostic factor

## Abstract

Invasive fungal infections (IFI) are emerging opportunistic diseases that occur mainly in immunocompromised patients. Our study aimed to analyze the epidemiology of IFIs in patients with hematological malignancies, and the prognostic factors. Our retrospective study included patients hospitalized in the hematology department between January 1^st^, 2010, and August 31^st^, 2020, and in whom the diagnosis of IFI was made according to the EORTC criteria 2008. We found 29 IFIs among 6989 admissions (0.4%). IFIs were proven in 16 cases and probable in 13 cases. The median age was 35 years. The sex ratio was 0.9. The predominant IFI was invasive pulmonary aspergillosis (n=14) followed by fungemia (n=13). Candida albicans was the most isolated species in blood cultures (5/9). The mortality rate was 48%. In multivariate analysis, disease status, time to start antifungal treatment, and lactate levels are significant factors of excess mortality. IFIs are responsible for significant morbidity and mortality. The challenge lies in the precocity of starting the treatment as well as the vigilance given to the factors of poor prognosis.

## Introduction

Invasive fungal infections (IFIs) are common opportunistic infections in immunocompromised patients, including patients with hematological malignancies [1,2]. They are responsible for significant morbidity and mortality. The most commonly encountered fungal infections are caused by *Candida albicans, Cryptococcus neoformans, Aspergillus fumigatus* and *Pneumocystis jirovecii*. However, there is an increase in the prevalence of infections caused by *Candida non-albicans, Aspergillus non-fumigatus*, but also by other emerging filamentous fungi such as zygomycetes (Mucorales), *Fusarium spp and Scedosporium spp* [1]. Due to the diversity of the epidemiological profile of IFIs found through studies, and the lack of information concerning the national status of these infections, it is therefore imperative to monitor the epidemiology of IFIs to be able to adopt the best first-line antifungal treatment. This work aims to analyze the epidemiology of IFIs in the hematology department of the military hospital of Tunis and to determine prognostic factors.

## Methods

**Study design and settings:** it is a retrospective study conducted in the laboratory of Parasitology-Mycology of the Military Hospital of Tunis in collaboration with the clinical hematology department of the same hospital over a period of 10 years and 8 months (January 1^st^, 2010-August 31^st^, 2020).

**Study population:** this study included 29 patients hospitalized in the clinical hematology department whose diagnosis of proven or probable IFIs was established by mycological examination in the laboratory of Parasitology-Mycology. IFIs were defined according to the EORTC/MSG (European Organization for Research and Treatment of Cancer-Mycoses Study Group) 2008 criteria.

**Laboratory analysis:** the identification of yeasts was made by the chlamydosporulation test, Auxacolor ® gallery or the YST Vitek®2 card. The identification of filamentous fungi was made according to macroscopic and microscopic criteria.

**Statistical analysis:** these data were analyzed using SPSS version 24 software. We studied the prognostic factors of IFIs by univariate and multivariate analysis. For all statistical tests, the significance level was p<0.05.

## Results

### Epidemiological characteristics

**Positivity rate:** among 6989 admissions, 29 patients presented an IFI. The positivity rate was 0.4% (4 episodes per 1000 admissions).

**Age distribution and sex ratio:** the mean age of our patients was 36.5 ± 16.6 years (extremes 11-74 years). The age group [17-32 years] was affected in 38% of cases followed by the age groups [49-64 years], [33-48 years], [0-16 years], and [≥65 years] in respectively 24%, 21%, 14%, and 3%. We noted a female predominance with a gender ratio of 0.9.

### Clinical characteristics

**Distribution of patients according to hemopathy:** the predominant underlying hemopathy was acute myeloid leukemia (AML) (n=16) followed by acute lymphoblastic leukemia (ALL) (n=6), Hodgkin and non-Hodgkin lymphoma (n=3), medullary aplasia (n=3), and multiple myeloma (n=1).

**Distribution of cases by hemopathy status:** forty-one percent of the patients were in relapse or disease progression (12/29 cases) at the time of diagnosis of IFIs. In 28% of the cases, IFIs occurred during the active phase of the hemopathy. On the other hand, we noted 6 cases of IFIs during complete remission of the hemopathy and 3 cases during partial remission.

**Distribution of cases according to IFIs:** invasive aspergillosis (IA) was the most frequent IFI, found in 48% of cases (n=14) followed by fungemia in 44% of cases (n=13), neuromeningeal cryptococcosis in 4% (n=1), and pulmonary mucormycosis in 4% (n=1). These infections were proven in 55% of cases and probable in 45% of cases based on the 2008 EORTC-MSG definition of IFIs.

**Distribution according to clinical, imaging, endoscopic signs and secondary locations during IFI:** the predominant clinical sign was fever (n=23; 79%) (Table 1). Chest CT scan images were suggestive of invasive pulmonary aspergillosis (IPA) in 10 cases and non-specific images in 4 cases. Seven patients developed secondary metastases (24%): cerebral, cardiac, cutaneous, hepatic and hepatosplenic.

**Table 1:** distribution of clinical signs according to each type of IFI

Type of IFI	Clinical signs	Number of patients
Invasive pulmonary aspergillosis	Fever	9/13
	Tachycardia	3/13
	Pleural pain	4/13
	Dyspnea	7/13
	Hemoptysis	3/13
	Other: costal swelling	1/13
Yeast fungemia	Fever	10/12
	Hypothermia	1/12
	Tachycardia	7/12
	Polypnea	4/12
	Chills	1/12
	Diarrhea	2/12
	Headache	2/12
Pulmonary mucormycosis	Fever, tachycardia, dyspnea, pleural pain, hemoptysis	1/1
Disseminated fusariosis	Fever, tachycardia, dyspnea and signs of struggle	1/1
Neuromeningeal cryptococcosis	Fever, headache, meningeal signs	1/1
Hepatosplenic aspergillosis	Fever, tachycardia, altered general condition	1/1

### Mycological characteristics

**Direct examination and culture:** the sensitivities of direct examination and culture for all samples combined were 61% and 89% respectively. Among the 13 blood cultures, yeasts of the genus Candida were identified in 9 cases, with a predominance of *Candida albicans* (n=5), followed by *Candida parapsilosis* (n=2), *Candida glabrata* (n=1), and *Candida tropicalis* (n=1). Two strains of *Trichosporon asahii*, one strain of *Geotrichum capitatum* and one strain of *Fusarium* s *pp* were isolated from blood cultures. *Aspergillus niger* was isolated in one BAL sample. *Cryptococcus neoformans* was isolated from a CSF sample. *Rhizomucor miehei* was isolated from a bronchial biopsy.

**Prognostic factors:** we noted a favorable evolution in 15 patients (52%). Fourteen patients died (48%). In univariate analysis: sex, disease status, duration of neutropenia, renal insufficiency, initiation of prophylactic antifungal treatment, time to initiation of antifungal therapy, fibrinogen level, procalcitonin level and lactate level were selected as poor prognostic factors in our study (Table 2). Multivariate analysis revealed that disease status (ORa 4.017; 95% CI 0.259-62.288), time to initiation of antifungal therapy (ORa 5.606; 95% CI 0.328-75.955) and lactate level (ORa 6.170; 95% CI 0.03-93.144) were significant factors for excess mortality.

**Table 2:** univariate analytical study of prognostic factors

Prognostic factors	Group with and without prognostic factor	Alive	Dead	P value
Type of IFI*	IA:14	7	7	0.573
	Fongemia:13	7	6	
Sex	Male:14	6	8	**0.043**
	Female:15	9	6	
Age	<65 years: 28	15	13	0.19
	≥65 years: 1	0	1	
Hemopathy	AML: 16	9	7	0.265
	ALL: 6	4	2	
	Lymphoma: 3	1	2	
	Medullary aplasia: 3	0	3	
	Multiple myeloma: 1	1	0	
Disease status	Complete remission: 6	4	2	**0.038**
	Partial remission: 3	3	0	
	Relapse/progression: 12	3	9	
	Active disease: 8	5	3	
Neutropenia	Yes: 27	13	14	0.259
	No: 2	2	0	
Depth of neutropenia	<500: 16	7	9	0.358
	500-1000: 1	1	0	
	>1000: 1	0	1	
Duration of neutropenia	≥3 weeks: Yes: 9	2	7	**0.041**
	No: 9	6	3	
Broad-spectrum Antibiotic therapy	Yes: 17	8	9	0.5
	No: 9	5	4	
Parenteral nutrition	Yes: 12	5	7	0.297
	No: 17	10	7	
Corticosteroid therapy	Yes: 8	6	2	0.155
	No: 20	9	11	
Renal failure	Yes: 4	15	10	**0.042**
	No: 25	0	4	
Extended stay in the hematology department	Yes: 20	9	11	0.25
	No: 9	6	3	
Central venous catheter	Yes: 11	6	5	0.558
	No: 18	9	9	
Prophylactic antifungal treatment	Yes: 9	3	6	**0.048**
	No: 17	10	7	
Time to start antifungal treatment (days)	Average for living: 0.67	15	-	**0.016**
	Average among decedents: 2.07	-	14	
Thrombocytopenia	Yes: 22	11	11	0.4
	No: 6	4	2	
Lactate level (mmol/l)	Average for living: 1.67	10	-	**0.035**
	Average among decedents:3.14	-	14	
Procalcitonin level (ng/ml)	Average for living:5.30	10	-	**0.043**
	Average among decedents:7.79	-	13	
Fibrinogen level (g/l)	Average for living:3.46	8	-	**0.048**
	Average among decedents:4.23	-	11	

* For the type of IFIs, neuromeningeal cryptococcosis and pulmonary mucormycosis were excluded from the list to avoid biasing the calculation.

## Discussion

During our study period, the positivity rate was 0.4%. The incidence of IFIs was 3 to 4% in onco-hematology patients [2]. The median age of our patients was 35 years old and the sex ratio was 0.9. Unlike, Fracchiolla *et al*. in a study of 196 onco-hematology patients who received antifungal treatment for IFIs, reported a sex ratio of 1.4 and a median age of 61 years [18-85 years] [3]. Regarding the underlying blood disease, we noted that AML was the most frequent followed by ALL. A study based on autopsy results confirmed that AML and myelodysplastic syndromes are the most common hematological malignancies associated with IFIs. Furthermore, high rates of proven invasive fungal diseases have recently been observed in patients with non-Hodgkin's lymphoma in the era of targeted drugs [4].

In our study, IFIs occurred in case of relapse/progression (41%) and during the active phase of the disease (28%). Hale *et al*. demonstrated, in univariate and multivariate analysis, that relapsed hematological malignancy is significantly associated with the development of IFI [5]. This can be explained by the use of more aggressive immunosuppressive treatments. Invasive pulmonary aspergillosis and candidemia were the main infections in our series. Indeed, aspergillosis, candidiasis, fusariosis, mucormycosis and cryptococcosis are the most frequently reported infections in patients with hematological malignancies [3]. The French RESSIF (*Réseau de Surveillance des Infections Fongiques*) Network data (2012-2018) showed a dominance of fungemia (49%) followed by pneumocystosis (20%), and invasive aspergillosis (15%) [6].

Fever was the main sign for all IFIs (79%) and was observed in 83% of yeast fungemia. Michel *et al*. demonstrated that the main clinical sign of candidemia was fever, which may be accompanied by poor hemodynamic tolerance or even septic shock [7]. In invasive aspergillosis, fever is often high and its association with pulmonary signs, chest pain, and hemoptysis is suggestive [7]. The sensitivity of direct examination varies according to sample types. The best sensitivity was noted in blood cultures (>50%). The analysis of the morphology of mycelial filaments on direct examination can orient the diagnosis of fungal species and thus allow the appropriate starting therapy. The fastest method for diagnosing cryptococcosis is direct microscopy of cerebrospinal fluid after Indian ink staining. However, its sensitivity varies from 30 to 50% in non-AIDS-related cryptococcal meningitis and up to 80% in AIDS patients [8].

*Candida albicans* remains an important pathogen as in our study, but an increasing rate of non-*albicans Candida* species is noted in many studies [2,3,9]. Montagna *et al*. reported in a prospective multicentre study, 27 IFIs among 589 onco-heamatology patients. Yeasts were isolated in 16 cases (59.2%) with predominance of the *Candida* genus (87.5%). *Cryptococcus neoformans* and *Geotrichum capitatum* (6.2%) were also isolated. Furthermore, a total of eleven mold infections (40.7%), 10 aspergillosis and 1 zygomycosis, were reported, with the isolation of 3 A. *fumigatus* and 1 *Rhizomucor pusillus* [9].

Other biological markers of poor prognosis have been reported, such as a monocyte count < 120/mm^3^ at diagnosis of IA, hyperbilirubinemia and renal failure [8]. Corticosteroid therapy ≥2 mg/kg of prednisone equivalent, uncontrolled GvH, and CMV disease have been described as poor prognostic factors [10].

The strength of our study is that being extended over a long period. In addition, rare Tunisian studies have focused on this alarming subject in recent years. The processing of samples in a uniform way by a single laboratory and according to a well-codified protocol shows a major interest in our monocentric study.

Nevertheless, some limitations of our work are: Being retrospective, our study was limited to data collected from patient records; the small number of cases diminished the performance of the statistical tests, and the absence of a control group allowing the identification of factors predisposing to IFIs represented a major difficulty.

## Conclusion

IFIs are responsible for significant morbidity and mortality in immunocompromised patients, especially those with hematological malignancies. Through this study, we contribute to the knowledge of the local epidemiology of IFIs in a Tunisian series: incidence, species involved, and prognostic factors. This could provide support to clinicians, allowing an optimization of recommendations for the treatment of IFIs.

### What is known about this topic

- *Invasive fungal infections (IFIs) are emerging opportunistic diseases that occur mainly in immunocompromised patients;*
- *Their epidemiological profiles are various through national and international studies;*
- *The epidemiological study of IFIs is imperative to adapt best first-line antifungal treatment*.

### What this study adds

- *This study is the first to be published concerning the epidemiological data about IFIs in Tunisia;*
- *This study contributes to the knowledge of the local epidemiology of IFIs over a long period of 10 years and 8 months;*
- *It also reports the analysis of prognostic factors of IFIs*.

## References

[1] Ibáñez-Martínez E, Ruiz-Gaitán A, Pemán-García J (2017). Update on the diagnosis of invasive fungal infection. Rev Esp Quimioter.

[2] Pagano L, Akova M, Dimopoulos G, Herbrecht R, Drgona L, Blijlevens N (2011). Risk assessment and prognostic factors for mould-related diseases in immunocompromised patients. J Antimicrob Chemother.

[3] Fracchiolla NS, Sciumè M, Orofino N, Guidotti F, Grancini A, Cavalca F (2019). Epidemiology and treatment approaches in management of invasive fungal infections in hematological malignancies: results from a single-centre study. PLoS One.

[4] Facchinelli D, Marchesini G, Nadali G, Pagano L (2018). Invasive fungal infections in patients with chronic lymphoproliferative disorders in the era of target drugs. Mediterr J Hematol Infect Dis.

[5] Hale KA, Shaw PJ, DallaPozza L, MacIntyre CR, Isaacs D, Sorrell TC (2010). Epidemiology of paediatric invasive fungal infections and a case-control study of risk factors in acute leukaemia or post stem cell transplant. Br J Haematol.

[6] Bretagne S, Sitbon K, Desnos-Ollivier M, Garcia-Hermoso D, Letscher-Bru V, Cassaing S, French Mycoses Study Group (2022). Active Surveillance Program to Increase Awareness on Invasive Fungal Diseases: the French RESSIF Network (2012 to 2018). mBio.

[7] Michel G (2011). Mycoses invasives au cours du traitement des maladies malignes de l´enfant. Arch Pediatr.

[8] Guarana M, Vidal JE, Nucci M (2018). Cryptococcosis in patients with hematologic diseases. CurrFungal Infect Rep.

[9] Montagna MT, Giglio OD, Napoli C, Lovero G, Caggiano G, Delia M (2012). Invasive fungal infections in patients with hematologic malignancies (aurora project): lights and shadows during 18-months surveillance. Int J Mol Sci.

[10] Robin C (2018). Infections fongiques compliquant les hémopathies et les greffes de cellules souches: incidence, facteurs de risque, mortalité, et développement de scores prédictifsdécisionnels pour améliorer la pertinence des prophylaxies [Thèse].

